# Supplementary material for: Genetic Diversity and Molecular Evolution of Hepatitis E Virus Within the Genus Chirohepevirus in Bats
Source: Viruses. 2025 Feb 28;17(3):339. doi: 10.3390/v17030339 (PMC11945734; doi:10.3390/v17030339)
Supplement: Supplementary file 1 [file viruses-17-00339-s001.zip › viruses-3502650-supplementary.pdf]

Table S1. GenBank accession numbers of the mitochondrial genomes of the bat species used in this study

Host family	Host species	GenBank accession no.
Rhinolophidae	*Rhinolophus ferrumequinum*	AB085731
Rhinolophidae	*Rhinolophus sinicus*	HM134917
Phyllostomidae	*Desmodus rotundus*	NC_022423
Vespertilionidae	*Eptesicus japonensis*	LC361451
Vespertilionidae	*Eptesicus serotinus*	MF187951
Vespertilionidae	*Miniopterus pusillus*	OR468083
Vespertilionidae	*Myotis davidii*	NC_025568
Vespertilionidae	*Myotis ricketti*	AB106608
Vespertilionidae	*Pipistrellus abramus*	AB085739
Vespertilionidae	*Pipistrellus nathusii*	AJ504446
Vespertilionidae	*Scotophilus kuhlii*	MT750321
Vespertilionidae	*Tylonycteris pachypus*	EF517313
Vespertilionidae	*Tylonycteris robustula*	ON640723

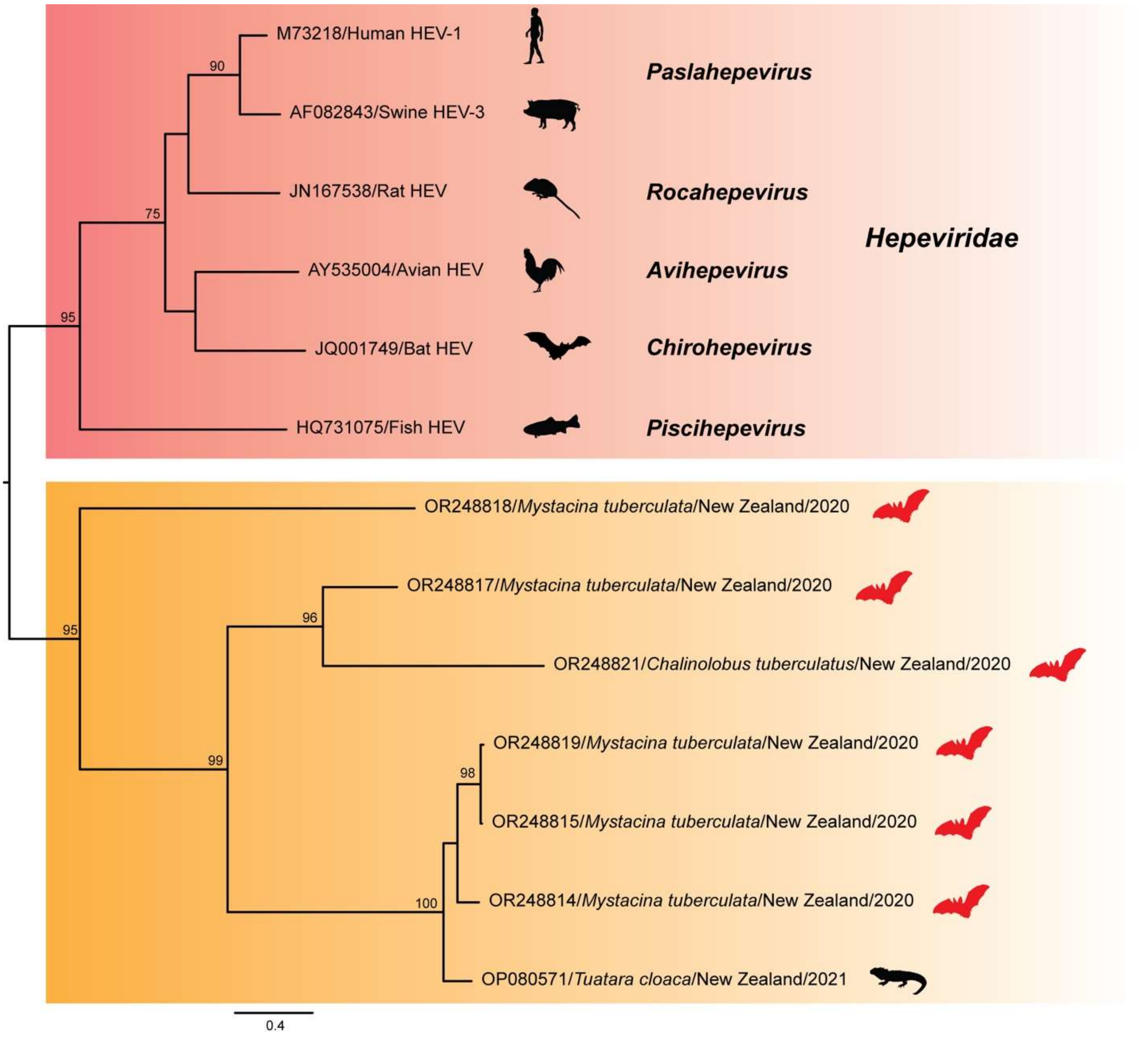

Figure S1. Maximum likelihood phylogeny of partial RdRp of HEV-related viruses. The partial RdRp region encompasses approximately 900 nucleotides. Virus designations include GenBank accession number and host species. Chirohepeviruses from New Zealand lesser short-tailed bats (*Mystacina tuberculata*) in the phylogenetic tree are highlighted in red. Bootstrap values of >70% are indicated at relevant nodes. Scale bar corresponds to the number of nucleotide substitutions per site.
